# A novel somatic mutation in ACD induces telomere lengthening and apoptosis resistance in leukemia cells

**DOI:** 10.1186/s12885-015-1639-5

**Published:** 2015-09-07

**Authors:** Jean-François Spinella, Pauline Cassart, Nicolas Garnier, Philippe Rousseau, Claire Drullion, Chantal Richer, Manon Ouimet, Virginie Saillour, Jasmine Healy, Chantal Autexier, Daniel Sinnett

**Affiliations:** 1 Division of Hematology-Oncology, Sainte-Justine UHC Research Center, 3175 Côte Ste-Catherine, H3T 1C5, Montréal, Québec Canada; 2 Lady Davis Institute Jewish General Hospital, Montreal, Qc Canada; 3 Departments of Anatomy, Cell Biology and Medicine, McGill University, Montreal, Qc Canada; 4 Department of Pediatrics, Faculty of Medicine, University of Montreal, Montreal, Qc Canada

## Abstract

**Background:**

The identification of oncogenic driver mutations has largely relied on the assumption that genes that exhibit more mutations than expected by chance are more likely to play an active role in tumorigenesis. Major cancer sequencing initiatives have therefore focused on recurrent mutations that are more likely to be drivers. However, in specific genetic contexts, low frequency mutations may also be capable of participating in oncogenic processes. Reliable strategies for identifying these rare or even patient-specific (private) mutations are needed in order to elucidate more personalized approaches to cancer diagnosis and treatment.

**Methods:**

Here we performed whole-exome sequencing on three cases of childhood pre-B acute lymphoblastic leukemia (cALL), representing three cytogenetically-defined subgroups (high hyperdiploid, t(12;21) translocation, and cytogenetically normal). We applied a data reduction strategy to identify both common and rare/private somatic events with high functional potential. Top-ranked candidate mutations were subsequently validated at high sequencing depth on an independent platform and *in vitro* expression assays were performed to evaluate the impact of identified mutations on cell growth and survival.

**Results:**

We identified 6 putatively damaging non-synonymous somatic mutations among the three cALL patients. Three of these mutations were well-characterized common cALL mutations involved in constitutive activation of the mitogen-activated protein kinase pathway (FLT3 p.D835Y, NRAS p.G13D, BRAF p.G466A). The remaining three patient-specific mutations (ACD p.G223V, DOT1L p.V114F, HCFC1 p.Y103H) were novel mutations previously undescribed in public cancer databases. Cytotoxicity assays demonstrated a protective effect of the ACD p.G223V mutation against apoptosis in leukemia cells. ACD plays a key role in protecting telomeres and recruiting telomerase. Using a telomere restriction fragment assay, we also showed that this novel mutation in ACD leads to increased telomere length in leukemia cells.

**Conclusion:**

This study identified ACD as a novel gene involved in cALL and points to a functional role for ACD in enhancing leukemia cell survival. These results highlight the importance of rare/private somatic mutations in understanding cALL etiology, even within well-characterized molecular subgroups.

**Electronic supplementary material:**

The online version of this article (doi:10.1186/s12885-015-1639-5) contains supplementary material, which is available to authorized users.

## Background

Childhood acute lymphoblastic leukemia is a heterogeneous disease both biologically and clinically, and is the leading cause of cancer-related deaths among children. Despite significant advances in our understanding of the pathobiology of cALL leading to risk-based treatment regimens and increased survival rates, the etiological causes of this disease remain elusive. Approximately 75 % of pre-B cALL cases exhibit hyperdiploidy or a recurring gross chromosomal rearrangement, detection of which is central to disease diagnosis, risk stratification and management [1]. While these chromosomal alterations play an important role in driving the leukemic process by affecting molecular pathways that halt lymphoid progenitor cell differentiation and promote cell proliferation and survival, they are not sufficient for leukemic transformation and are often detected years before leukemia onset. This suggests a need for additional cooperating events in order to achieve overt leukemogenesis [2]. Thorough investigation of cALL genomes is crucial to better understand the underlying genomic complexity of this disease and thus better diagnose and treat it. Toward these goals, recent large-scale sequencing efforts revealed many somatic driver mutations/genes recurrently mutated in cALL [1]. The identification of these high frequency driver mutations is essential to better understand disease etiology and characterize prognostic subgroups. Yet accumulating evidence has also shown that low-frequency mutations within a cancer type can contribute to onset and progression of the disease [3], and play a role in intra-tumor and inter-patient heterogeneity. The identification of patient-specific mutations could provide crucial information regarding molecular pathways underlying cALL tumorigenesis and thus point to new therapeutic avenues. Here, we performed whole-exome sequencing of 3 pre-B cALL cases. Case 1 was diagnosed with high hyperdiploiy (>50 chromosome) cALL and Case 2 harboured the t(12;21) (*ETV6-RUNX1*) translocation. Together these two molecularly defined subgroups represent over 40 % of cALL cases. Case 3 was cytogenetically normal at diagnosis. Using a unique quartet design, we sequenced matched normal (blood following remission) and tumor (bone marrow at diagnosis) patient samples, and the parents of each case. We successfully identified known recurrent drivers, as well as novel patient-specific somatic mutations with high functional potential. Using *in vitro* assays, we showed that the private p.G223V mutation, adjacent to the TEL patch of the telomere protein ACD (also known as TPP1), leads to apoptosis resistance and may contribute to leukemia cell survival by promoting telomere maintenance and protection.

## Methods

### Patients

All study subjects were self-declared French-Canadians of European descent from the established Quebec cALL (QcALL) cohort [4]. The Sainte-Justine UHC Research Ethics Board approved the protocol. Informed consent was obtained from the parents of the patients to participate in this study and for publication of this report and any accompanying images. A copy of the written consent is available for review by the Editor of this journal.

Case 1, a 10 year old male, was classified as high risk based on his age. He presented with a platelet count of 15.0 × 10^9^/L, a white blood cell count (WBC) of 9.0 × 10^9^/L, and 63 % and 97 % lymphoblast cells in the blood and bone marrow samples respectively. The cytogenetic analysis revealed high hyperdiploidy (karyotype: 53,XY,+3,+4,+6,+10,+14,+17,+18). He was enrolled on the Dana Farber Cancer Institute (DFCI) ALL Consortium protocol 95–01. He achieved complete remission and has been out of treatment for over 60 months with leukemia free-survival (LFS).

Case 2 was a 4 year old female with a platelet count of 35.0 × 10^9^/L, a WBC of 33.1 × 10^9^/L, and 75 % and 96 % lymphoblast cells in the blood and bone marrow samples respectively. Cytogenetic analysis revealed a t(12;21) translocation. Despite her karyotype, usually associated with a good prognostic, she was classified as high risk based on the presence of leukemia cells in the cerebrospinal fluid. She was enrolled on DFCI/ALL Consortium protocol 2000–01. She also achieved complete remission and has been out of treatment for over 60 months with LFS.

Case 3 was a 6 year-old male with a WBC of 183.1 × 10^9^/L, a platelet count of 29.0 × 10^9^/L, 87 % and 95 % lymphoblast cells in the blood and bone marrow samples respectively, and presented with a normal karyotype according to the cytogenetic analysis. The patient was classified as high risk based on hyperleukocytosis and was treated on DFCI/ALL Consortium Protocol 2000–01. Case 3 experienced a first relapse 41 months post diagnosis, and following 2 subsequent relapses he died 73 months post diagnosis.

### Whole exome sequence capture and sequencing

**Fig. 1:**
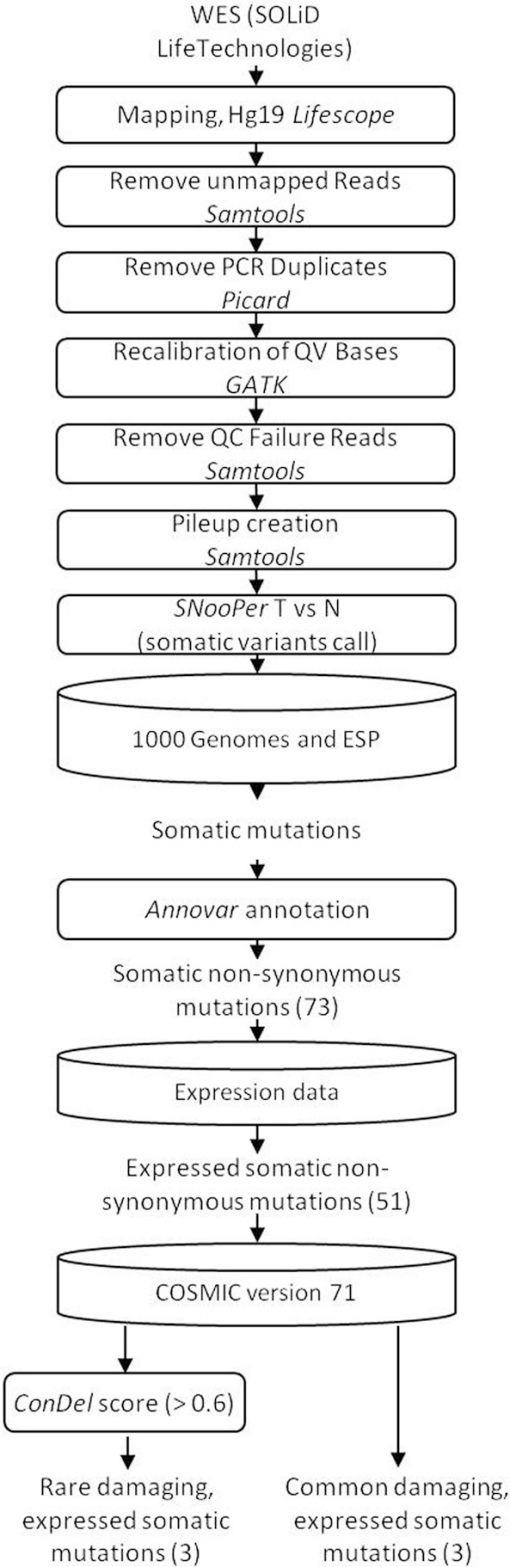
Whole-exome sequencing analysis workflow. Boxes represent the analysis/cleaning steps. Cylinders represent the SNV filtering steps used in the data reduction strategy to identify functional somatic mutations. The number of variations remaining after each step is shown in brackets. Note that only SNVs that passed a given filter were tested for in the subsequent step. Using public databases and variant annotation tools, we identified 6 top-ranked mutations among the pre-B cALL patients, including 3 SNVs referenced in COSMIC v71 and 3 candidate rare/private SNVs in ACD (p.G223V), DOT1L (p.V114F) and HCFC1 (p.Y103H) with a potential functional impact

DNA was extracted from bone marrow samples (at diagnosis) and peripheral blood samples (obtained after remission) from the cases and their parents using standard protocols as described previously [5]. Whole exomes were captured in solution with Agilent’s SureSelect Human All Exon 50 Mb kits for SOLiD sequencing (Life Technologies) according to the manufacturer’s protocol, were sequenced on the Life Technologies SOLiD System (mean coverage =32X) and aligned to the Hg19 reference genome using LifeScope Genomic Analysis Software (see Fig. 1 for complete sequencing analysis workflow). Polymerase chain reaction (PCR) duplicates were removed using Picard [http://broadinstitute.github.io/picard/]. Base quality score recalibration was performed using the Genome Analysis ToolKit (GATK) [6] and reads that failed the quality control were removed. Cleaned BAM files were used to create pileup files using SAMtools [7]. Somatic single nucleotide variants (SNVs) were called from pileup files using SNooPer, an in-house variant caller that is based on a machine learning approach that integrates tumoral and normal data and that was specifically trained for optimal identification of somatic mutations in our low-depth SOLiD sequencing data (manuscript submitted and software available upon request). Furthermore, using the familial design, we were able to use parental sequence information to remove Mendelian inconsistencies, reduce false-positive sequencing and alignment errors and facilitate somatic variant identification. The robustness of this approach was further demonstrated using high-depth sequencing on an independent platform to confirm top-ranked somatic mutations (see below). Resulting somatic SNVs were queried against publically available datasets such as 1000 Genomes [8] and NHLBI GO Exome Sequencing Project (ESP) [http://evs.gs.washington.edu/EVS/] to filter out common polymorphism eventually remaining (minor allele frequency >0.01).

### Expression data filter

Publically available microarray expression data [9] were used to filter variants based on the assumption that expressed genes are more likely to carry functionally relevant mutations [10]. Mutated genes identified from each case were compared to the expression profiles of the corresponding cALL subgroup (hyperdiploidy, t(12;21), or “others”) and only expressed genes were subsequently retained in the somatic variant analysis.

### Ultra-deep targeted re-sequencing

Top-ranked rare/private candidate mutations, as well as common somatic mutations identified in the pre-B cALL cases were validated on the Ion Torrent system. Selected variant regions (~125pb flanking the identified somatic mutation) were amplified and PCR products were sequenced on the Ion PGM Sequencer (Life Technologies) according to the manufacturer’s protocol with a mean coverage >1,800X. Primer sequences and PCR protocols are available upon request.

### Site-directed mutagenesis and apoptosis assay

Identified mutations were introduced by site-directed mutagenesis into the complementary DNA (cDNA) sequence of each gene cloned into a pDONR221 vector. Using the pLenti-CMV-DEST Gateway Vector (w118-1), we subcloned the wild-type (WT) or mutant coding sequences and generated lentiviruses using a Third Generation Packaging System in 293 T cells. Lentiviral particles were then harvested and used to infect Nalm-6 (human leukemia pre-B cells) with 8ug/mL polybrene. Infected cells were selected with puromycin 1 μg/μL, seeded at 5 × 10^5^ cells/mL in their culture medium and treated with 5 μM of camptothecin for 3 h. Apoptosis was measured using conventional Annexin V/Propidium iodide (PI) staining and quantified by flow cytometry (BD Biosciences FACS Fortessa). All experiments were done in triplicate.

### Telomere restriction fragment (TRF) assay

Parental Nalm-6 cells and Nalm-6 cells overexpressing mutant ACD p.G223V (Nalm-6 ACD G223V), the wild-type ACD (Nalm-6 ACD WT) or Nalm-6 cells infected with the empty vector alone (Nalm-6 pLENTI) were used for TRF assays. For each cell line, extracted DNA at passage 16 (p16) (population doublings ≈ 50) and p27 (population doublings ≈ 80) after selection was digested with HinfI and RsaI restriction enzymes and fractionated on an electrophoresis gel apparatus. After drying, the gel was hybridized with a [γ -32P] adenosine triphosphate (ATP)end-labelled (T2AG3)3 probe and exposed on X-ray film. Mean TRF length was calculated as previously described [11]. The TRF assay was performed in duplicate.

## Results/discussion

Assuming that functionally important genes can also be mutated more rarely and in specific tumor contexts, we performed whole-exome sequencing of three pre-B cALL patients and their parents and applied a data reduction strategy to identify both common and novel rare/private events with high functional potential (Fig. 1). SNVs were queried against public databases, annotated using ANNOVAR [12], and were binned according to frequency and function (Methods). Non-synonymous mutations that were expressed in cALL subgroups (based on microarray cALL expression data [9]) were then filtered based on a CONsensus DELeteriousness score >0.6 (measure of the degree of coherence of individual methods about the likelihood that a SNV is deleterious) [13]. This led to the identification of 6 expressed damaging non-synonymous somatic mutations among the 3 patients (FLT3 p.D835Y, NRAS p.G13D, BRAF p.G466A, ACD p.G223V, DOT1L p.V114F, HCFC1 p.Y103H) (Table 1) that were subsequently confirmed using targeted ultra-deep re-sequencing (mean coverage >1800X). Among these 6 somatic SNVs, FLT3 p.D835Y, NRAS p.G13D and BRAF p.G466A were referenced in the Catalogue Of Somatic Mutations In Cancer (COSMIC) database (v71) and previously shown to constitutively activate the mitogen-activated protein kinase (MAPK) pathway and to increase tumor proliferation [14–17]. The identification of previously reported driver mutations validates the robustness of our approach. The NRAS p.G13D gain of function mutation was identified in Case 1, which corroborates previous studies that report postnatal RAS activating mutations in ~30 % of pre-B hyperdiploid cALL patients [18]. The BRAF p.G466A mutation identified in Case 2 is associated with a mild increase of ERK activation [19]. While mutations in BRAF are identified in almost 70 % of patients with multiple myeloma [15], screening of childhood pre-B cohorts only identified a few cases harbouring these events [20]. Although alterations of the receptor tyrosine kinase FLT3 are frequent in hyperdiploid cALL and rare in other subtypes [21], FLT3 p.D835Y was identified in the cytogenetically normal Case 3. While Case 3 suffered a relapse and activated forms of FLT3 are usually associated with a poor outcome in acute myeloid leukemia patients [22], this association is not confirmed in cALL [23].

**Table 1:** Candidate somatic mutations identified in each patient

Patient	Subgroup	Gene	Genomic change	Protein change	Class	VAF	COSMIC v71
Case 1	HD	NRAS	g.chr1:115258744C > T	p.G13D	Missense	0.48	Haematopoietic and Lymphoid tissue
		ACD/TPP1	g.chr16:67693443C > A	p.G223V	Missense	0.51	-
		DOT1L	g.chr19:2191086G > T	p.V114F	Missense	0.46	-
Case 2	t(12;21)	BRAF	g.chr7:140481411C > G	p.G466A	Missense	0.15	Thyroid
		HCFC1	g.chrX:153230064A > G	p.Y103H	Missense	0.51	-
Case 3	CN	FLT3	g.chr13:28592642G > T	p.D835Y	Missense	0.44	Haematopoietic and Lymphoid tissue

Variant allele frequencies (*VAF*) (number of supporting reads/coverage) were calculated based on ultra-deep targeted re-sequencing data (mean coverage >1800X). Only tissue types harbouring the highest occurrence of the mutation in the COSMIC database (v71) are presented. *HD* hyperdiploid, *CN* Cytogenetically normal

Our sequencing analysis pipeline also led to the identification of three candidate novel pathogenic mutations in Cases 1 and 2 (DOT1L p.V114F, HCFC1 p.Y103H, ACD p.G223V), that were neither previously reported nor referenced in public databases. Based on our strict filtering criteria, no private mutations were identified in Case 3. DOT1L is a histone writer and HCFC1 is a broad transcription regulator that plays a critical role in cell proliferation via its involvement in chromatin-modifying activities [24]. These results are consistent with recent cancer sequencing initiatives that highlighted the important role of chromatin remodeling genes in leukemogenesis [25]. Furthermore, DOT1L has recently been implicated in the development of MLL-rearranged leukemia and shown to be essential for leukemic transformation [26]. On the other hand, ACD is a core protein in the shelterin complex and mediates the access of telomerase to the telomere. It is essential for telomere homeostasis in hematopoietic stem and progenitor cell particularly [27, 28].

**Fig. 2:**
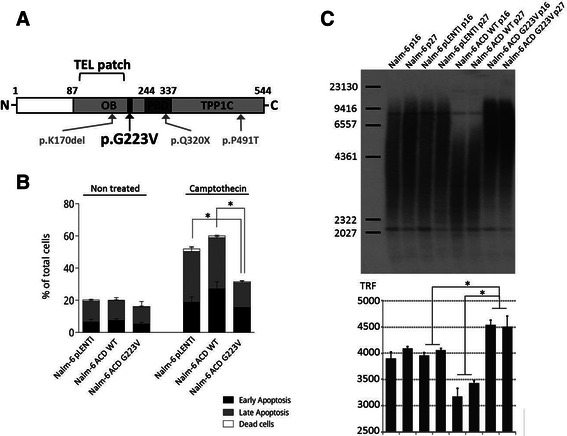
ACD p.G223V protects from camptothecin-induced apoptosis and increases telomere length. **a**. Schematic representation of the ACD protein. The p.G223V mutation, depicted in black, is adjacent to the TEL patch of the OB-fold domain involved in telomerase recruitment and composed of seven critical amino acids located in a region defined by the curly bracket (E168, E169, E171, R180, L183, L212 and E215) [30]. p.Q320X, p.P491T and p.K170del, depicted in grey, are three germline mutations recently identified and associated with familial melanomas and bone marrow failure disorders [34–36]. TPP1C corresponds to the TIN2-binding domain. Together, the OB (oligonucleotide/oligosaccharide-binding) and PBD (POT1 binding domain) domains form the ACDN domain necessary for POT1 binding to telomeric DNA and the stimulation of telomerase processivity. **b.** *In vitro* apoptosis assays show overall reduced levels of apoptosis associated with ACD p.G223V. The c.659 g > t mutation was introduced into the ACD transgene by site-directed mutagenesis and expressed in Nalm-6 cells. The graph shows annexin V/PI staining for 3 h on Nalm-6 pLenti (empty vector), Nalm-6 ACD WT and Nalm-6 ACD G223V cells. **c.** The telomere restriction fragment assay (TRF) showed a quantitative increase in telomere size for Nalm-6 ACD G223V cells at passage 16 (p16) and p27 after selection. Mean TRF length = ∑ (ODi)/∑ (ODi/Li) where ODi is the radioactive signal, Li is the TRF fragment length at position i. The bar chart of Fig. 2c (bottom) represents the mean TRF length for each condition directly quantified from each corresponding lane of the TRF gel presented at the top of Fig. 2c Significance (in **b** and **c**) was determined by a Mann–Whitney *U* test; *p*-values <0.05 are represented by an asterisk

The high variant allele frequency (VAF) of these rare/private mutations (DOT1L, VAF =0.46; HCFC1, VAF =0.51; ACD, VAF =0.51), calculated based on our ultra-deep targeted re-sequencing data (Table 1), was indicative of early clonal selection supporting a possible functional role in leukemia development. Functional validation of the ACD p.G223V mutation (Fig. 2a) was further pursued using *in vitro* expression assays, however our *in vitro* lentiviral expression system did not permit functional characterization of neither DOT1L nor HCFC1, due to the size of the open reading frames (ORFs) (4.6 kb and 6.1 kb, respectively) that were beyond the viral packaging capacity of the capside [29]. Further investigation of these two novel mutations using alternative functional screening methods is ongoing. *In vitro* expression assays of ACD using the topoisomerase I inhibitor camptothecin, showed that leukemic cells overexpressing mutant p.G223V ACD (Nalm-6 ACD G223V) exhibit lower levels of apoptosis compared with cells overexpressing wild-type ACD (Nalm-6 ACD WT) (*P* =0.014, Mann–Whitney *U* test) and the empty vector (Nalm-6 pLenti) (*P* =0.014, Mann–Whitney *U* test) (Fig. 2b). The p.G223V mutation is adjacent to the TEL patch in the oligonucleotide/oligosaccharide-binding (OB) fold domain of ACD that interacts directly with the catalytic subunit of telomerase. Within the shelterin complex, ACD was specifically shown to interact with POT1 to protect telomeres and recruit telomerase at chromosome ends [30–32]. Interestingly, recurrent somatic mutations in the OB domains of POT1 have been shown to cause telomere dysfunction in chronic lymphocytic leukemia suggesting that alteration of shelterin-mediated protein-telomere binding could lead to genomic instability and cancer [33]. Furthermore, very recently, germline mutations in the POT1 binding domain and the TEL patch of ACD were respectively associated with familial melanomas and bone marrow failure disorders [34–36] (Fig. 2a). Although further studies are needed to decipher the underlying molecular mechanisms implicating ACD in apoptosis inhibition, taken together, these data strongly support a role for ACD p.G223V in promoting leukemia cell maintenance.

To further investigate the effect of p.G223V mutant ACD on telomere structure, we performed a TRF assay (Fig. 2c) and showed that decreased apoptosis correlates with increased telomere length in Nalm-6 ACD G223V at both tested passages compared to Nalm-6 ACD WT and Nalm-6 pLenti (*P* =0.029, Mann–Whitney *U* test), confirming stable alteration of telomere homeostasis. ACD mutant induced telomere elongation in leukemia cells is consistent with reports demonstrating disrupted shelterin complex function and telomere lengthening due to mutations in POT1 in chronic lymphocytic leukemia [33, 37]. Furthermore, as observed for POT1 at the same population doubling point [33], overexpression of WT ACD did not increase telomere length (Fig. 2c), confirming that the observed telomere lengthening is due to the p.G223V mutation and not caused by global overexpression of ACD. Concomitant lengthening of the telomeres and decreased apoptosis levels in NALM-6 ACD G223V following camptothecin treatment corroborates previous findings that altered telomerase activity can lead to hypersensitivity of tumor cells to topoisomerase inhibitors [38, 39]. Further investigation is required to characterize the influence of the p.G223V mutation on the recruitment and processivity of telomerase and on telomere-length regulation. Missense or frameshift mutations in ACD, many of which are located in the OB fold, adjacent to the TEL patch, and in the POT1 interaction domain, have been described in multiple cancer types (Additional file 1 Table S1) with the highest prevalence found in melanoma (2.7 %), suggesting that ACD mutations may participate in a common underlying cancer-promoting pathway that involves telomere dysfunction. Case 1 who carries the ACD p.G223V mutation had high hyperdiploid cALL and although telomere dysfunction can cause chromosome destabilization and aneuploidy [40, 41], it is unlikely that this mutation precluded the mitotic event producing the hyperdiploid phenotype. However, our results do support a role for ACD p.G223V in the earlier stages of clonal expansion contributing to telomere maintenance and apoptosis resistance, at least *in vitro*. Further investigation is required in order to characterize the mechanistic role of ACD p.G223V within this patient’s specific tumor context.

## Conclusions

The prenatal origins of cALLs are well established, along with the need for additional postnatal mutations in order to drive overt leukemogenesis [42, 43]. The extent to which rare/private genetic events contribute to the onset and progression of cALL however is largely unknown. Through whole-exome sequencing of 3 cALL cases, we successfully identified not only common drivers (NRAS p.G13D, FLT3 p.D835Y, and BRAF p.G466A), but also rare/private somatic mutations (DOT1L p.V114F, HCFC1 p.Y103H, ACD p.G223V), in well-characterized cALL molecular subgroups. The identification of patient-specific events with a functional potential is not surprising and further confirms the underlying genetic complexity of this disease. We went on to demonstrate the functional impact of ACD p.G223V on apoptosis resistance and telomere-length regulation in pre-B ALL cells. The high VAF of this somatic mutation suggests that it is likely present in the major subclone; while this does not necessarily imply functionality, it does support an early role for ACD p.G223V in driving the leukemic process. Though further investigation is needed to fully characterize the influence of the identified mutation on telomere homeostasis, this study is the first to describe the functional implications of a somatic mutation in ACD on leukemic cell behaviour, supporting a role for ACD and telomere regulation in leukemia cell resistance to apoptosis. In conclusion, these results support the need of thorough investigation of rare/private mutations to reveal the underlying complexity of cALL landscapes, including within well-characterized subgroups, and the inter-patient variability that may influence diagnosis and prognosis.

### Consent

The Sainte-Justine UHC Research Ethics Board approved the protocol. Informed consent was obtained from the parents of the patients to participate in this study and for publication of this report and any accompanying images. A copy of the written consent is available for review by the Editor of this journal.

## Additional file

Additional file 1: Table S1. Description of data: Non-synonymous and frame-shift mutations in ACD referenced in the public cancer database COSMIC version 71. (PDF 120 kb)

## Abbreviations

cALL: childhood acute lymphoblastic leukemia
WBC: White blood cell count
DFCI: Dana Farber Cancer Institute
LFS: Leukemia free-survival
GATK: Genome Analysis ToolKit
SNVs: Somatic single nucleotide variants
ESP: NHLBI GO Exome Sequencing Project
TRF: Telomere restriction fragment
COSMIC: Catalogue of Somatic Mutations In Cancer
VAF: Variant allele frequency
ConDel score: CONsensus DELeteriousness score
PCR: Polymerase chain reaction
WT: Wild-type
PI: Propidium iodide
OB: Oligonucleotide/oligosaccharide-binding fold domain
PBD: POT1 binding domain
TPP1C: TIN2-binding domain
HD: hyperdiploid
CN: Cytogenetically normal
