# Supplementary material for: A novel somatic mutation in ACD induces telomere lengthening and apoptosis resistance in leukemia cells
Source: BMC Cancer. 2015 Sep 7;15:621. doi: 10.1186/s12885-015-1639-5 (PMC4562123; doi:10.1186/s12885-015-1639-5)
Supplement: Additional file 1: Table S1. — Description of data: Non-synonymous and frame-shift mutations in ACD referenced in the public cancer database COSMIC version 71. (PDF 120 kb) [file 12885_2015_1639_MOESM1_ESM.pdf]

A novel somatic mutation in TPP1 induces telomere lengthening and apoptosis resistance in leukemia cells.

Supplementary Table 1. Non-synonymous and frame-shift mutations in TPP1 referenced in the public cancer database COSMIC version 71.

CDS change	Protein change	COSMIC v71 ID	Count	Class	Domain
c.79G>A	p.G27R	COSM3670197	1	Missense	-
c.100C>T	p.R34*	COSM1519517	1	Nonsense	-
c.125C>T	p.A42V	COSM4141846	1	Missense	-
c.151C>T	p.L51F	COSM3511076	3	Missense	-
c.154C>A	p.L52I	COSM972546	2	Missense	-
c.167C>T	p.P56L	COSM3888782	1	Missense	-
c.181C>T	p.P61S	COSM141471	2	Missense	-
c.184C>T	p.L62F	COSM135719	2	Missense	-
c.187C>T	p.P63S	COSM3888781	1	Missense	-
c.188C>T	p.P63L	COSM3511075	1	Missense	-
c.221A>G	p.N74S	COSM4061942	1	Missense	-
c.229C>G	p.P77A	COSM1479001	1	Missense	-
c.257G>T	p.G86V	COSM4061941	1	Missense	-
c.283C>G	p.L95V	COSM119718	1	Missense	OB
c.294G>A	p.W98*	COSM703839	1	Nonsense	OB
c.335C>T	p.P112L	COSM3511074	1	Missense	OB
c.505T>G	p.F169V	COSM3932389	1	Missense	OB
c.559C>T	p.H187Y	COSM140768	1	Missense	OB
c.562G>A	p.V188I	COSM972544	1	Missense	OB
c.739G>A	p.A247T	COSM1302137	1	Missense	PBD
c.794A>G	p.Q265R	COSM4061939	1	Missense	PBD
c.865delC	p.H289fs*30	COSM972542	1	Frameshift	PBD
c.866A>G	p.H289R	COSM3370509	1	Missense	PBD
c.1054C>A	p.P352T	COSM3511073	1	Missense	TPP1C
c.1066C>A	p.P356T	COSM417319	1	Missense	TPP1C
c.1096G>A	p.G366S	COSM4061938	1	Missense	TPP1C
c.1109_1110CC>TT	p.S370F	COSM143470	1	Missense	TPP1C
c.1141C>T	p.P381S	COSM972540	1	Missense	TPP1C
c.1208G>A	p.C403Y	COSM1378975	1	Missense	TPP1C
c.1214C>T	p.A405V	COSM471951	1	Missense	TPP1C
c.1231C>A	p.P411T	COSM349226	1	Missense	TPP1C
c.1244A>C	p.H415P	COSM3932388	1	Missense	TPP1C
c.1253G>A	p.R418H	COSM1378974	1	Missense	TPP1C
c.1286C>T	p.P429L	COSM3511072	1	Missense	TPP1C
c.1301G>A	p.R434H	COSM1740245	1	Missense	TPP1C
c.1337C>T	p.T446I	COSM1749706	2	Missense	TPP1C
c.1396C>T	p.R466W	COSM1378973	1	Missense	TPP1C
c.1399C>T	p.P467S	COSM4061937	1	Missense	TPP1C
c.1400C>T	p.P467L	COSM1378972	1	Missense	TPP1C
c.1430G>A	p.G477E	COSM417320	1	Missense	TPP1C
c.1455G>T	p.W485C	COSM4061936	1	Missense	TPP1C
c.1474C>T	p.R492C	COSM1644358	1	Missense	TPP1C
c.1603G>T	p.G535W	COSM972538	1	Missense	TPP1C

TPP1C: TIN2-binding domain; OB: oligonucleotide/oligosaccharide-binding; PBD: POT1 binding domain.
